# Progress in Medicine

**Published:** 1899-05-27

**Authors:** 


### Progress in Medicine.

### DISEASES OF THE STOMACH.

(*Continued from page* 131.)

**Hyperchlorydria.**—Olivetti[21] reports on his results with Fleiner's plan of treatment by giving two and a half to five drachms of bismuth nitrate every morning through a tube for perhaps three weeks. He finds great relief of the pain and discomfort is produced, though there is little change in the secretions or movements of the stomach which Fleiner claims for it. Pick[22] describes a condition where the stomach is hypersensitive to food and other stimuli, so that severe pain follows eating. True hyperchlorydria may sometimes accompany the state. Fluids are as irritating as solids, acids particularly so. The pain is diffuse, the vomit contains chiefly fluid, and the heartburn commences at once on the ingestion of food. The gastric secretions are healthy, and the treatment should aim at curing any neurosis present and regulating the diet. Magnesia may be given to allay the heartburn. Gaston Lyon[23] lays down some valuable rules on the treatment of hyperchlorydria. He insists on abstinence from tobacco and the disuse of aperients. Undue mental exertion and excesses in diet must be prevented. In severe forms, with either periodic or continuous over-secretion, he finds that rest in bed, with a strict milk diet and lime water, as well as cold compresses to the stomach, are of use. If pain continues then alone it is desirable to give drugs, bismuth and magnesia, but not soda or the alkaline waters of Vichy. Relapses may be easily caused by alcohol or tobacco. In the continuous form milk diet and rest are all important, to be followed by a diet in which vegetables and starchy food are given in very limited quantities only. Lavage with pure water may do good if not used too often or too long.

**Nervous Dyspepsia** is shown, according to L. Herzog,[24] by a definite group of symptoms of chronic type. Thus we often find motor insufficiency with hyperacidity leading later on to a normal or subnormal acidity. It is rare to find purely sensory changes, but if the motor power remains good variations of acidity may be present, and even in the empty stomach disturbances may arise. Grace Murray[25] notices the great variations in acidity in this disorder, together with the eructations and vertigo, which appear to her specially frequent, as well as nausea, though actual vomiting is rare. She would not include in the group the dyspepsias arising from reflex irritation, such as uterine and ocular troubles. The stomach condition in the gastric crises of locomotor ataxia have been examined by several authors. Douglas,[26] in three cases, found that the amount of free H.Cl. was small; the total acidity was not more than that in health, and that blood appeared several times in the vomit. In a later paper[27] he gives detailed analyses, and concludes that during the crises the stomach pours out a quantity of fluid slightly inferior to that in health, and without any excess of acid or H.Cl. The blood clearly cannot come from an ulcer, for between the crises the digestion may be painless and perfect, and nothing of the kind has been found post-mortem. It seems to him possible that the bleeding may come from engorged veins. J. Michell Clark[28] found in a case of his an absence of H.Cl. during the crises, together with a large amount of lactic acid. In the intervals there was rapid digestion and an excess of H.Cl. after meals.

**Cancer of the Stomach.**—Cheney[29] records no less than 20 cases in patients under thirty years of age and 17 others below forty, thus showing that cancer at these ages is not so rare as is thought. His patients numbered 150 in all, and the prominent symptoms were pain in 130, vomiting in 128, a tumour in 115, hæmaturesis in 36, and severe dyspepsia in 38. In the course of diagnosis inflation of the stomach by means of soda and tartaric acid is of great use and quite safe in the opinion of the author. The Oppler-Boas bacillus has been investigated recently by Knickerbocker,[30] who concludes that, though not pathognomonic of cancer, it is an important sign, and may be found at some stage in nearly all cases. In some it is discoverable before a tumour appears, and in many it may be seen so early that extirpation is practicable. Stenosis of the pylorus, though sometimes the result of ulcer or cancer, is not infrequently due to spasm from other causes. In such cases it would be of the highest importance to avoid operative interference if possible. Dr. Poulet[31] successfully treated a case by the injection of hyoscyamine and morphia. These were given every evening, and in a month there was a practical disappearance of all bad symptoms. The affection had existed for a long period, and caused violent suffering, but the patient was able afterwards to return to his ordinary mode of life. A. Robin[32] advises in cases of stenosis of doubtful origin complete rest in bed, with a pure milk diet in small divided quantities every two hours. If the patient fails to gain weight steadily after the first week surgical measures will be necessary. It may be needful to wash out the stomach at first if the dilatation has been extreme, but this should not be continued for more than a short time.

**Chronic Catarrh.**—Caporate[33] finds benefit from irrigating the stomach with weak solutions of zinc sulphate. In four patients whose illness lasted over a year the results were satisfactory. Lavage, with solutions of a strength gradually increased from gr. 1½ to gr. 12 to the quart was performed when the stomach was empty, and was followed by irrigation with a weak solution of sodium bicarbonate in order to convert any remaining sulphate into the harmless carbonate. This was done daily three times a week, and complete cures with a steady increase of weight were obtained.

An allied affection of long duration is described by Reichman.[34] There is complete absence of gastric juice, together with nausea, regurgitation of a watery fluid of a salty taste and alkaline reaction, which is apparently derived from the mucous glands of the stomach. There is also a twisting abdominal pain usually near the umbilicus which precedes the regurgitation. These attacks come on usually in the night of alternate days, and are regarded by Reichman as indicative of true atrophic gastritis, though their absence must not be regarded as counter-indicating the disease. Lockhart Gillespie[35] points out that many persons treated for stomach disorders are really suffering from intestinal sepsis. Hence their symptoms rapidly disappear when milk alone is given in small quantities with calomel to stop bacterial action in the duodenum and jejunum, creasote for the ileum, and salol for the rest of the intestine. He would later on also cut off malt liquors and strong wines, and regulate the diet not on any hard and fast lines, but with regard to the individual. Thus, it is often desirable to begin with a dose of calomel and a saline purge, to be followed by a small quantity of salol twice daily. Simon[36] finds that in gastric catarrh with deficient acid very remarkable effects are produced by giving small daily doses of sulphate of soda. Some 10 or 15 grains in hot water are sipped every morning, the ordinary diet being permitted except fat meat and acids. The use of the Glauber's salts in a few days removes all the distressing symptoms, and regulates the bowels, even in persons who have been ill for years. Little or no effect was seen, on the other hand, in persons who suffered from nervous dyspepsia, gastrectasis, atrophic gastritis, or troubles due to tubercle or cancer. Chittenden[37] finds that alcohol not only increases the amount of saliva, but also its power of digesting starch. The amount of hydrochloric acid is also increased, and probably the pepsin. Alcohol, moreover, is so rapidly absorbed from the stomach that probably none enters the intestine. In the test tube one or two per cent. of absolute alcohol does not interfere with the action of pepsin upon proteids, but with 18 per cent. it is greatly retarded. This represents a state of things where one-third of the stomach contents are pure whisky, but such an amount would be quickly reduced by absorption, while the water being unabsorbed by the stomach the percentage would, of course, fall rapidly. However, ordinary spirits, beer, wine, and particularly sherry, retard digestion far more than would be the case with corresponding amounts of pure alcohol. This is also true of claret when in large doses. Pancreatic digestion would be more seriously affected by alcohol, but it is not probable that it gets into the small intestines. Salivary digestion is also much retarded by the presence of alcohol in the stomach, particularly when it is in the form of wines, on account of the acids they contain. Thus delicate persons with weak digestion should avoid especially acid wines and vinegar as a regular article of diet on account of their retarding influence on digestion. Chittenden holds the view that alcohol is oxidised in the body and retards the oxidisation of proteids, besides causing an increased formation and excretion of uric acid. The food value of a wineglassful of 20 per cent. alcohol is about the same as one of starch, as regards the energy liberated.

[21] Brit. Med. Jour., Dec. 24. [22] Ibid. [23] Treatment, Jan. 12. [24] Med. Rec., Jan. 21. [25] Med. Rec., Feb. 4. [26] Glas. Med. Jour., Feb. [27] Lancet, April 15. [28] Brit. Med. Jour., Dec. [29] Int. Med. Jour., Jan. [30] Post Graduate, Mar. [31] Les Nouveaux Remédes, Feb. [32] Brit. Med. Jour., Dec. 12. [33] Jour. Amer. Med. Assoc., Jan. 21. [34] Brit. Med. Jour., Feb. 11. [35] Edin. Med. Jour., Nov. [36] Treatment, Jan. 12. [37] Mercks Archives, Feb.

